# Supplementary material for: Comparative Genomic and Expression Analysis Insight into Evolutionary Characteristics of PEBP Genes in Cultivated Peanuts and Their Roles in Floral Induction
Source: Int J Mol Sci. 2022 Oct 17;23(20):12429. doi: 10.3390/ijms232012429 (PMC9604132; doi:10.3390/ijms232012429)
Supplement: Supplementary file 1 [file ijms-23-12429-s001.zip › FiguresS1.pdf]

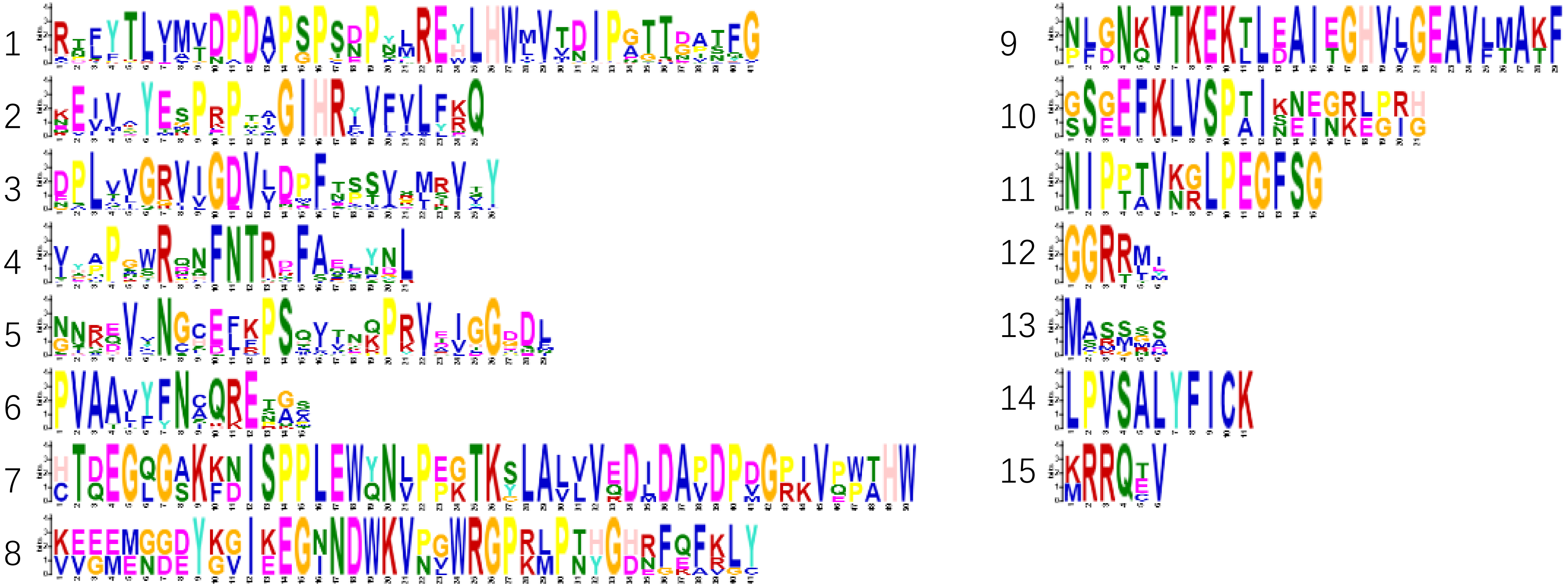
1
2
3
4
5
6
7
8
9
10
11
12
13
14
15

Figure S1. PEBP proteins from A. hypogaea were found to have motif sequences. The MEME software is used to do phantom analysis. Repeatability is shown by the height of distinct amino acids. The length of the motif protein sequence is indicated by the scale bar at the bottom
